# Analysis of synonymous codon usage of transcriptome database in *Rheum palmatum*

**DOI:** 10.7717/peerj.10450

**Published:** 2021-01-04

**Authors:** Xiaowei Huo, Sisi Liu, Yimin Li, Hao Wei, Jing Gao, Yonggang Yan, Gang Zhang, Mengmeng Liu

**Affiliations:** 1 College of Pharmaceutical Science, Institute of Life Science and Green Development, Hebei University, Baoding, China; 2 Hunan Academy of Forestry, Changsha, China; 3 College of Pharmacy and Shaanxi Provincial Key Laboratory for Chinese Medicine Basis & New Drugs Research, Shaanxi University of Chinese Medicine, Xi’an, China; 4 College of Traditional Chinese Medicine, Hebei University, Baoding, China

**Keywords:** Codon usage bias, Nucleotide composition, Transcriptome analysis, Gene expression level, Rheum palmatum

## Abstract

**Background:**

*Rheum palmatum* is an endangered and important medicinal plant in Asian countries, especially in China. However, there is little knowledge about the codon usage bias for *R. palmatum* CDSs. In this project, codon usage bias was determined based on the *R. palmatum* 2,626 predicted CDSs from R. palmatum transcriptome.

**Methods:**

In this study, all codon usage bias parameters and nucleotide compositions were calculated by Python script, Codon W, DNA Star, CUSP of EMBOSS.

**Results:**

The average GC and GC3 content are 46.57% and 46.6%, respectively, the results suggested that there exists a little more AT than GC in the *R. palmatum* genes, and the codon bias of *R. palmatum* genes preferred to end with A/T. We concluded that the codon bias in* R. palmatum* was affect by nucleotide composition, mutation pressure, natural selection, gene expression levels, and the mutation pressure is the prominent factor. In addition, we figured out 28 optimal codons and most of them ended with A or U. The project here can offer important information for further studies on enhancing the gene expression using codon optimization in heterogeneous expression system, predicting the genetic and evolutionary mechanisms in* R. palmatum*.

## Introduction

Codon degeneracy is a common phenomenon among living beings as the 20 different amino acids are encoded by 64 types of codons. Most amino acids encoded by two to six codons except for Met or Trp (Duret, 2002; Ang et al., 2016), this phenomenon is defined as synonymous codon. It has been reported that the using frequency of synonymous codon in encoding amino acids is non-random for different genes or genomes, which is regarded as codon usage bias (Romero, Zavala & Musto, 2000). The formation of codon bias can be influenced by multiple forces, such as natural selection, mutational pressure, and random genetic drift (Bentele et al., 2013). Moreover, the codon usage bias may affect gene transcription, protein expression and some other biological processes of protein expression (Behura & Severson, 2013; Powell & Dion, 2015). Performing the codon usage bias analysis and identifying the characteristics of codon usage bias for certain genes or genomes are very significant for comprehending the molecular mechanisms of gene expression and for understanding the long-term molecular evolution in genomes.

*Rheum palmatum* is an endangered and very important medicinal plant in China. The dried root and rhizomes of *R. palmatum* (named rhubarb) have been used to treat various diseases like infectious disease, cancer, constipation, and renal disorders in Asian countries (Zalucki, Power & Jennings, 2007). It has been reported that the main bioactive compounds of *R. palmatum* are anthraquinones (Jang & Kuk, 2018). *R. palmatum* is mainly distributed in northwest China, and the wild resources of *R. palmatum* are decreasing rapidly due to the overexploitation of natural resources (Zhou et al., 2014; Wang et al., 2016b). Therefore, it is beneficial for us to understand the anthraquinones biosynthetic pathways based on exploring the codon usage characteristics of *R. palmatum* and so to protect the wild resources of *R. palmatum*.

The genomic information of *R. palmatum* is not determined now. RNA-seq technology is an effective method to introduce transcriptome database, which can provide large numbers of coding sequences (CDS) with non-coding and repetitive sequences excluded. The transcriptome of *R. palmatum* was assembled with the RNA-seq method and 140,224 unigenes were screened out in our previous study. In this project, we carried out a series of analysis to determine the codon usage patterns of *R. palmatum* and pointed out the optimal codons. The results are intelligible for us to unify the molecular evolution of *R. palmatum* and provide some new perspectives to decipher gene function and carry out genetic engineering of *R. palmatum* in the future.

## Materials & Methods

### Database preparation

The transcriptome database of *R. palmatum* used in this project was obtained from our previous research work due to the lack of genomic information. In total, 140,224 annotated unigenes were obtained to analysis the coding DNA sequences (CDS) using the ESTScan (Iseli, Jongeneel & Bucher, 1999) and BLASTx online software (https://blast.ncbi.nlm.nih.gov/Blast.cgi). 14917 CDS were left after analysis by using ESTScan and BLASTx online software. Among these CDSs, the sequences contain correct initiation and termination codons were remained and sequences contain internal termination codons were eliminated by using SelectCDS script (Meier et al., 2017). In additional, only the sequences longer than 100 amino acids in length were hold for further analysis. Finally, 2,626 CDS were left for the downstream codon usage analysis.

### Indices of codon usage and codon bias

GC3s is an important parameter for evaluating the frequency of guanine + cytosine at the third synonymously coding position, excluding Met, Trp, and termination codons. The value of RSCU (Relative Synonymous Codon Usage) was calculated by dividing the observed frequency of codon usage by that expected under the situation that all codons encoding the same amino acid are used in the same probability. When the value of RSCU is great than 1 suggests the positive codon bias, and RSCU is smaller than 1 indicates the negative codon bias, whereas the value of RSCU equals 1 that means the codons to be used randomly or equally (Zhang et al., 2017). The values of ENc (Effective Number of Codons) range from 20 to 61, that are used to estimate the codon bias for an individual gene. An ENc value equals to 20 indicates that the amino acid in genes encoded by only one codon, while an ENc value equals to 61 indicates that the absence of codon usage preference (Sharp & Li, 1987). The study has shown that when the ENc value smaller than 36, the gene is affirmed to own strong codon usage preference (Fuglsang, 2006). CAI (Codon Adaptation Index) was often used to evaluate the extent of bias toward codons that were known to be preferred in highly expressed genes. The values of CAI range from 0 to 1.0, and the bigger the value is meaning the most frequently used synonymous codon (Fuglsang, 2008). CAI is calculated using the CAI calculate server (http://genomes.urv.es/CAIcal/).

### Neutrality plot analysis

The neutrality plot (GC12 vs GC3) can demonstrate the balance between mutation and selection in shaping codon bias. GC12 represents the average ratio of GC content in the first and second position of the codons (GC1 and GC2), while GC3 stands for the GC content in the third position. If we found a statistically strong correlation between GC12 and GC3, we can suggest that the dominant driving force for the *R. palmatum* is mutation pressure. Conversely, if there is no correlation between GC12 and GC3, we can see that the dominant driving force for the *R. palmatum* is natural selection (Sharp & Li, 1987; Sueoka, 1988).

### ENc-GC3s plot and PR2-Bias plot analysis

The ENC-GC3s (ENC vs GC3s) plot is usually carried out to exam the codon usage of a certain gene is only affect by mutation or also by other terms such as natural selection. The ENc-GC3s plot is constructed by the abscissa GC3s values and the ordinate ENC values. Furthermore, we calculate an expected curve on the ENc-GC3s plot. If we discover that the corresponding points distribute around expectation curve, we can conclude that the mutation pressure is the independent force in the progress of forming codon bias. If the corresponding points is significantly blow or far from the expected curve, there must be some other factors such as natural selection plays a key role in the formation of codon bias (Sueoka, 1988).

We have obtained the values of frequency of each nucleotide at the third site of codon (A3, U3, C3 and G3) and then performed the Parity Rule 2 bias (PR2-Bias) plot [A3/ (A3 + U3) vs G3/ (G3 + C3)] analysis (Wang et al., 2016a).

### Correspondence analysis of codon usage

Correspondence analysis (COA) is a widely accepted approach to determine the multivariate statistical analysis of codon usage patterns. As the genes possess 59 sense codons (61 sense codons in all, but exclude the unique Met and Trp codons), we put all the genes into a 59-dimensional hyperspace in the plot. The method can explore the major trend in codon usage variation among *R. palmatum* CDS and distributes the codons in axes with these trends based on RSCU values (Sueoka & Kawanishi, 2000).

### Determination of optimal codons

We get the top and bottom 5% of genes based on the CAI values as the high and low dataset, respectively. Then we get the mean RSCU values of the two gene groups, respectively. Finally, we carried out the Chi-squared contingency test to confirm the optimal codons. The usage frequency of certain codons that was remarkably higher in genes with high expression levels than that genes with low expression levels (*p* < 0.01) were regarded as optimal codons (Sau et al., 2005).

## Results

### Nucleotide composition of *R. palmatum* and codon bias

**Figure 1:**
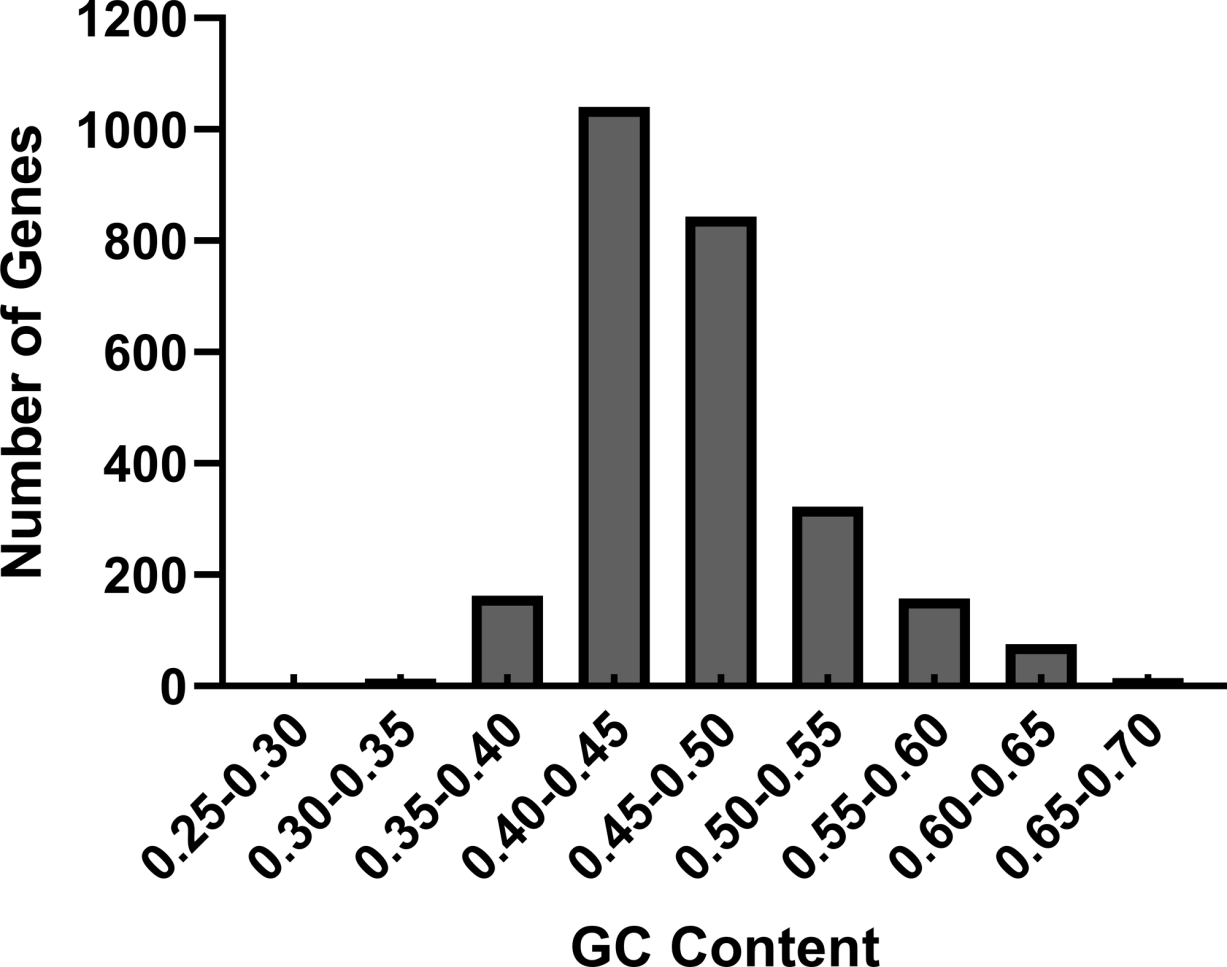
The distribution of GC contents in the CDS of *R. palmatum*.

We performed the nucleotide composition of 2,626 CDS in *R. palmatum* as the nucleotide content can affect the codon bias seriously (Nie et al., 2014). Among the 2,626 CDS in *R. palmatum*, the nucleotide content of A varies from 1.58% to 58.90%, with a mean value of 30.42%, the frequency of T is 5.89%–43.89%, with an average value of 23.48%, the proportion of G is 6.52%– 52.44%, with a mean value of 21.03%, the ratio of C is 4.49%–53.27%, with a mean value of 24.46%. To further understand the impact of nucleotide contents of *R. palmatum* genes on codon bias, we also calculated the GC contents and GC3. The results demonstrated that the GC contents of all genes ranged between 40% and 50% (Fig. 1). The mean value of GC proportion was 46.57%, which indicated that the genes in *R. palmatum* are richer in AT contents than that of GC. The ratio of GC1, GC2 and GC3 was 51.33%, 41.38 and 46.60, respectively, which testified that the proportion of GC1 was highest, and the contents of GC2 was very similar to that of GC3.

**Figure 2:**
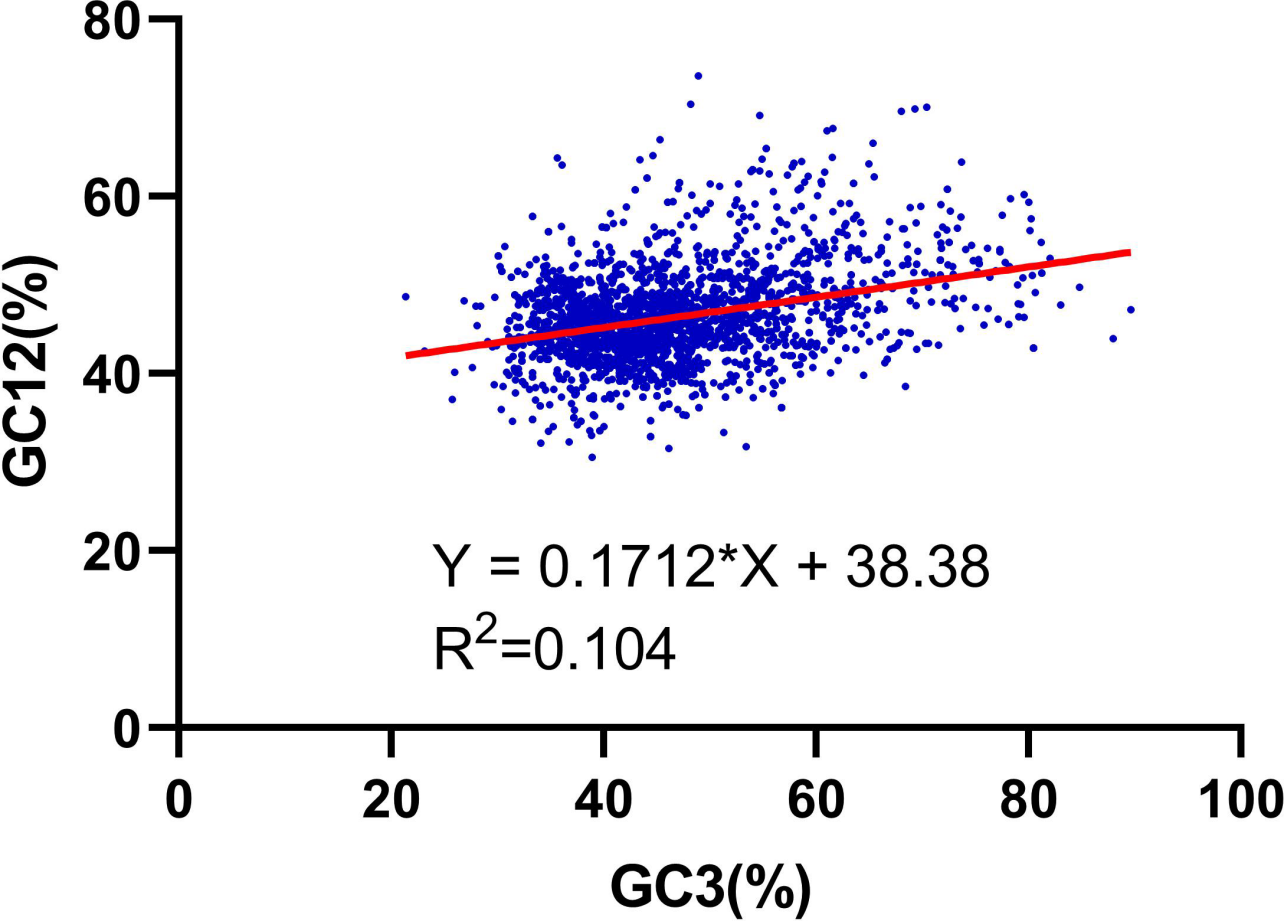
Neutrality plot analysis of the GC12 and that of the third codon position (GC3) for the entire coding DNA sequence of *R. palmatum*.

We further performed the relationship analysis between GC12 and GC3 using the neutrality plots (GC12 against GC3) method. The content value of GC3 varied from 21% to 89%. The results shown that the slopes of the regression lines (regression coefficient) were 0.171 (Fig. 2), verifying that the dominant force of the codon bias was natural selection in the RNA-seq data of *R. palmatum*, rather than mutation pressure.

### The effect of RSCU and ENC on codon bias

The ENC values in *R. palmatum* range from 30.83 to 61.00, with a mean value of 52.83. From the ENC values we can see that only 9 genes show a high codon bias with an ENC value smaller than 35. The results suggested that there is a common random codon usage in *R. palmatum* genes, without a strong codon bias preference. In addition, the RSCU values of 59 sense codons also indicates that the genes in *R. palmatum* are with a weak codon bias preference. As shown in the Table 1, more than half of the codons (30/59), marked in bold, are used frequently.

**Table 1:** Codon usage of *R. palmatum* genes.

AA	Codon	RSCU	AA	Codon	RSCU	AA	Codon	RSCU	AA	Codon	RSCU
**Phe**	UUU	**1.07**	**Ser**	UCU	**1.5**	**Tyr**	UAU	**1.1**	**Cys**	UGU	0.32
	UUC	0.93		UCC	0.93		UAC	0.9		CGC	0.57
**Leu**	UUA	0.7		UCA	**1.27**	**His**	CAU	**1.27**		CGA	0.64
	UUG	**1.43**		UCG	0.52		CAC	**1.98**		CGG	0.58
	CUU	**1.42**	**Pro**	CCU	**1.49**	**Gln**	CAA	**1.52**		AGA	**1.76**
	CUC	0.92		CCC	0.69		CAG	**1.42**		AGG	**1.69**
	CUA	0.6		CCA	**1.25**	**Asn**	AAU	**1.26**	**Ser**	AGU	0.89
	CUG	0.93		CCG	0.57		AAC	0.94		AGC	0.89
**Ile**	AUU	**1.31**	**Thr**	ACU	**1.31**	**Lys**	AAA	**1.18**	**Gly**	GGU	**1.12**
	AUC	0.92		ACC	0.9		AAG	**1.63**		GGC	0.79
	AUA	0.77		ACA	**1.26**	**Asp**	GAU	**1.21**		GGA	**1.23**
**Val**	GUU	**1.47**	**Ala**	GCU	**1.55**		GAC	0.61		GGG	0.85
	GUC	0.76		GCC	0.82	**Glu**	GAA	**1.18**			
	GUA	0.65		GCA	**1.17**		GAG	**1.24**			
	GUG	**1.12**		GCG	0.46						

**Notes.**

The preferentially used codon s are displayed in bold.

### The role of GC3s in the codon bias formation

**Figure 3:**
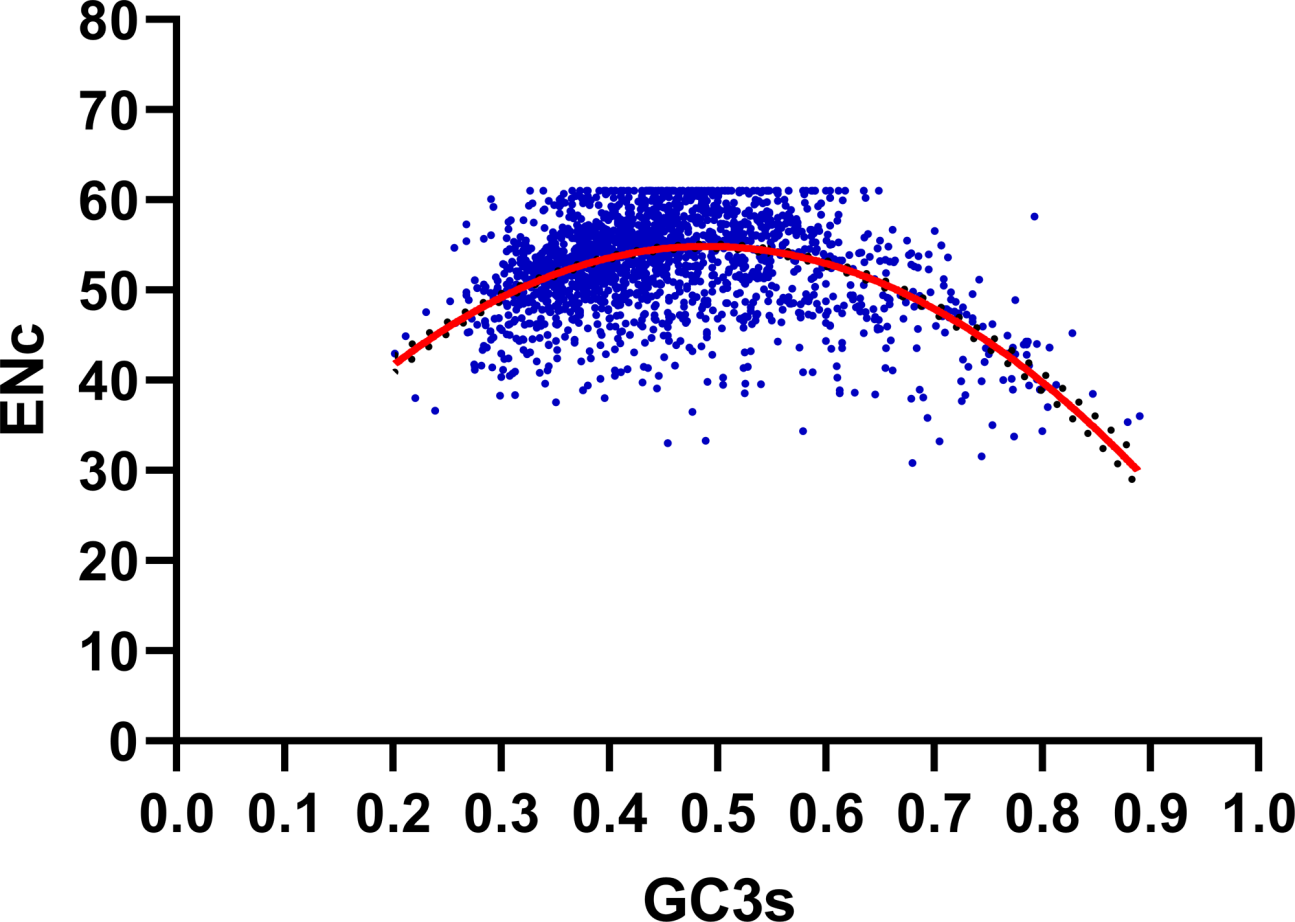
ENC-GC3 plot. The solid line represents the expected curve when codon usage bias is only affected by mutation pressure.

We performed the ENC-plot here to distinguish the influence of GC3s in shaping codon bias of *R. palmatum.* As we can see from the Fig. 3, most of the *R. palmatum* genes were distributed far from the expected ENC-plot curve, only a few genes are were distributed around this curve. The result suggested that the mutational pressure is not the only factor in shaping the codon bias, other factors such as translational selection may play an important role in the formation of codon bias.

**Figure 4:**
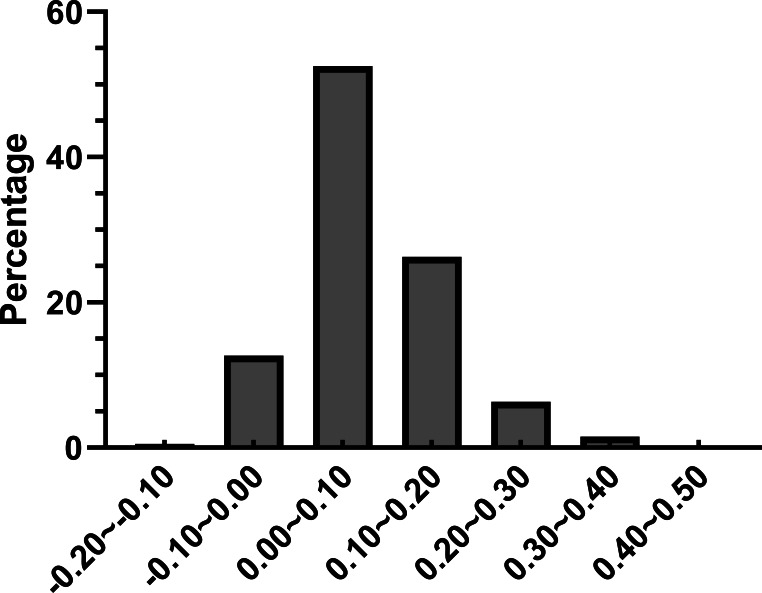
Frequency distribution of (ENCexp-ENCobs)/ENCexp.

Further we calculated (ENCexp-ENCobs)/ENCexp for all the genes in *R. palmatum* to discriminate the difference between observed and expected ENC values (Sinclair & Choy, 2002). As shown in Fig. 4, (ENCexp-ENCobs)/ENCexp values for most genes were within 0.00–0.10 which indicated that the most expected ENC values were bigger than actual ENC values. The values of GC3s affected the results of ENC value according to the calculation formula of expected ENC. The distribution frequency of (ENCexp-ENCobs)/ENCexp values was consistent with the results that have been shown in Fig. 3, which suggested that the different values of GC3s can affect the result of codon bias. Taken together, the results provide more evidences that GC3s play as a conditional mutational bias.

### Correspondence analysis

**Figure 5:**
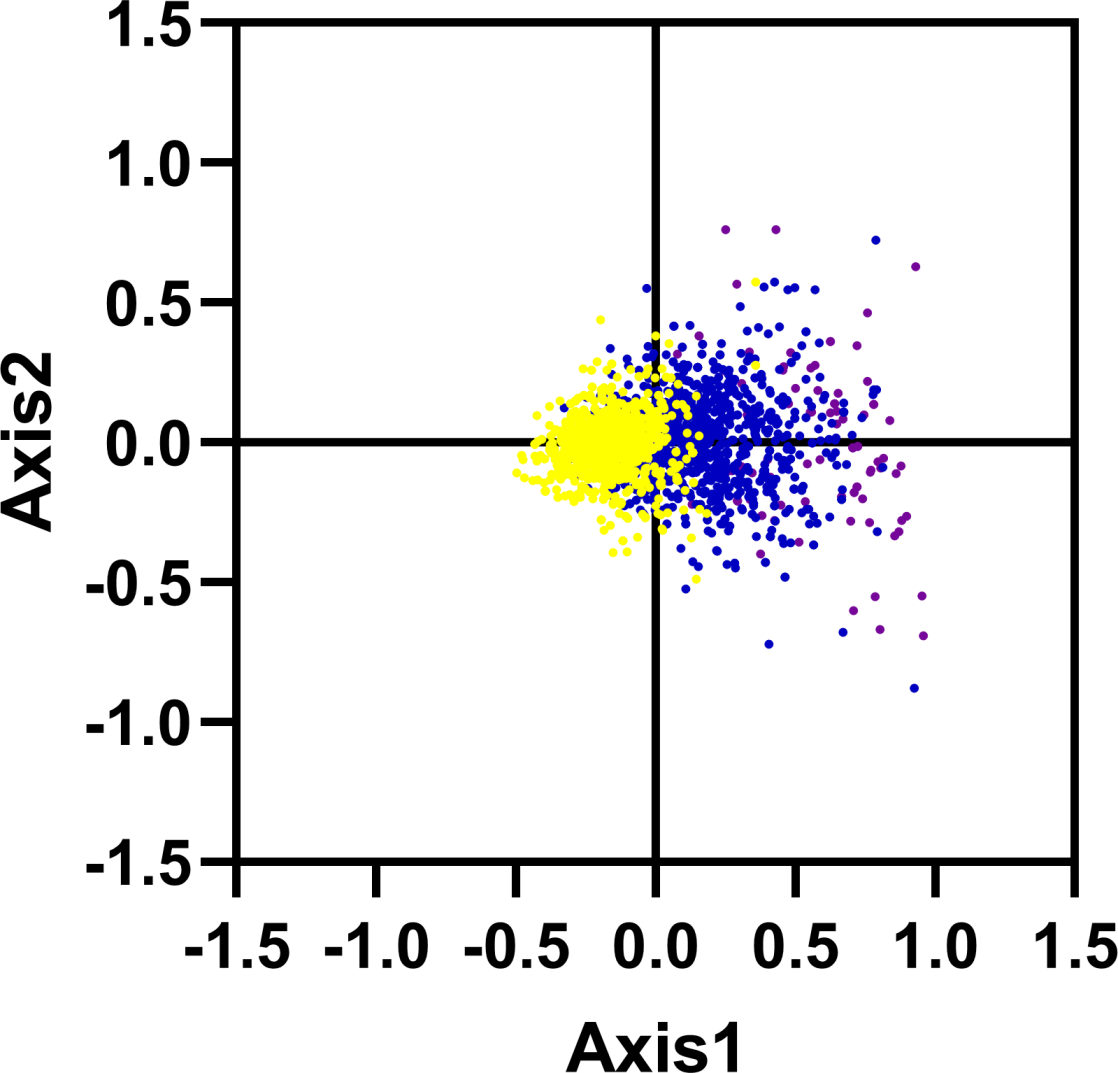
Correspondence analysis of codon usage bias: genes with GC content higher than 60%, within 45%–60% and lower than 45% were plotted as red, blue and green dots, respectively.

We carried out the correspondence analysis based on the RSCU values of all genes from *R. palmatum*. The result has shown that the Axis 1 and Axis 2 were two main contributors which contribute 11.53% and 6.38% of the total variance, respectively. As shown in Fig. 5, the position of the genes on the plane defined by the first two axes. Moreover, to check the role of GC content in shaping codon bias, the GC content of genes was higher than 60% marked with purple color, the GC content was between 45% and 60% marked with blue color, the GC content was below 45% marked with yellow color, respectively. As shown in Fig. 5, the red dots were separated along the primary axis, the blue and green dots located in the middle of the plot.

In addition, we have calculated the correlations among the six important parameters including Axis 1, GCall, GC3, GCall, ENC and CAI (Table 2). The parameter Axis 1 exhibited significant correlations with other four parameters such as GC12, GC3, GCall and CAI (*r* = 0.458, *r* = 0.931, *r* = 0.687, *r* = 0.260; *p* <  0.001), indicating that the codon bias of *R. palmatum* genes were influenced by two main factors including mutational pressure and translational selection.

**Table 2:** Correlation analysis of *R. palmatum* gene-related parameters.

Parameters	GC3	GCall	ENC	CAI	Axis1
GC12	0.327^**^	0.677^**^	−0.095^**^	0.059^**^	0.458^**^
GC3		0.654^**^	−0.053^*^	0.232^**^	0.931^**^
GCall			−0.093^**^	0.176^**^	0.687^**^
ENC				−0.054^**^	0.025
CAI					0.260^**^

**Notes.**

* Significant difference at *p* < 0.05.

** Significant difference at *p* < 0.001.

### C/T to A/G balance analysis in third codon

**Figure 6:**
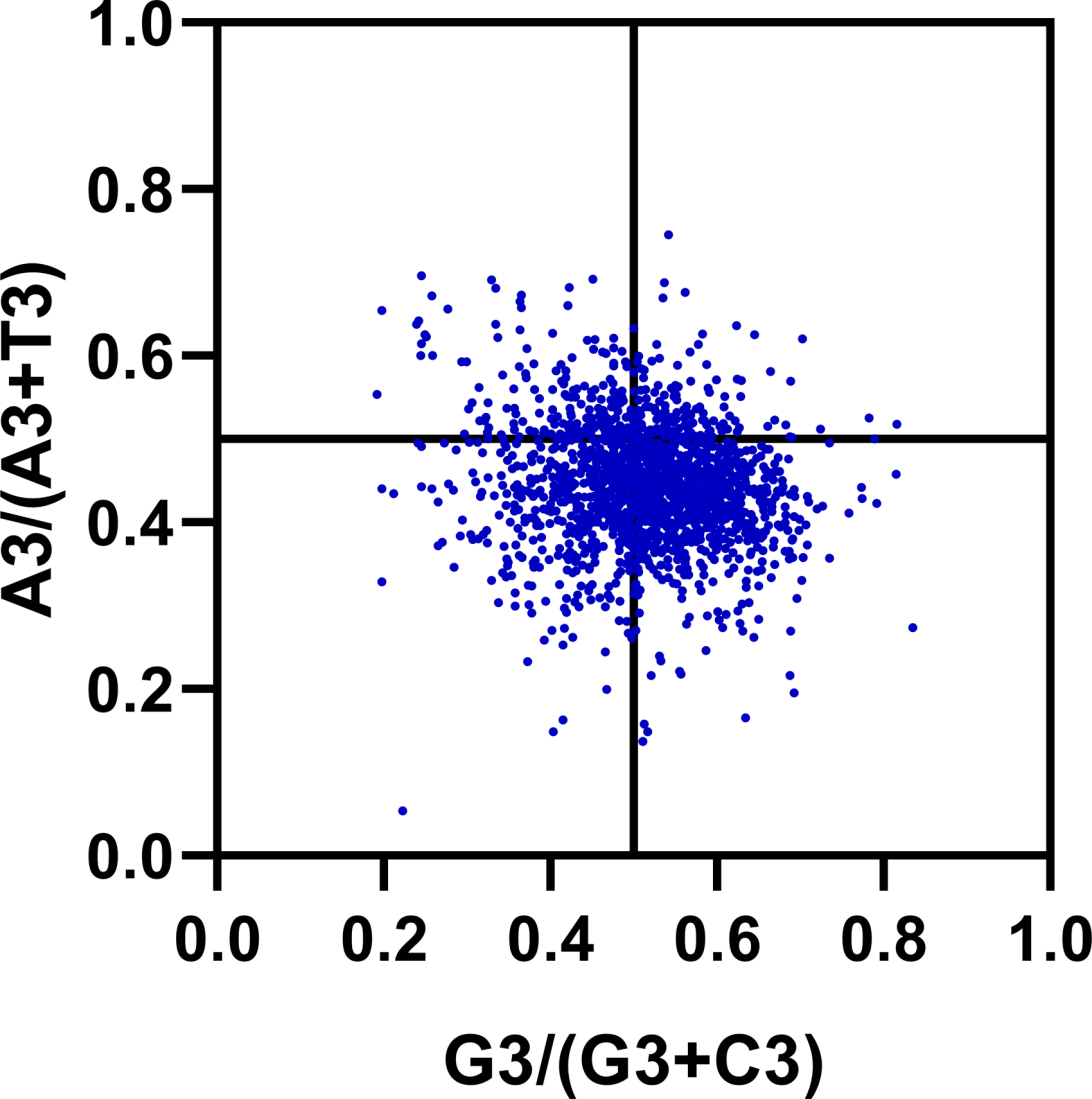
PR2-bias plot. Using the values of A3/(A3 + U3) against G3/(G3 + C3).

The previous studies about the effect of mutation pressure on codon bias have been reported that AU and GC come in pairs at third codon positions (Wright, 1990). However, our results suggest that the frequency of A3 and U3 (or G3 and C3) are different in *R. palmatum* genes. Estimating the proportion of GC and AT pairs in genes can offer more details about the effect of the forces on the codon bias formation. We carried out a Parity Rule 2 (PR2) plot analysis to determine whether there exist biases *R. palmatum* genes. As shown in Fig. 6, most of the points were distributed between 0.2 and 0.8 in the plot which suggesting that there is a low bias in either G3/C3 or A3/T3 in *R. palmatum*. In addition, the plot was divided into four quadrants taking 0.5 as the center on both axes. We found that there are more points in the fourth quadrant (the ratio of G3/GC3 and A3/AT3 > 0.5) than other three quadrants. The second quadrant contained the fewest points. All the results obtained above suggested that there is a slight significant preference for A and G at the third codon position of the *R. palmatum* genes. Therefore, there were some other forces like translational selection playing roles in the formation of codon bias.

### Effects of gene expression level

**Figure 7:**
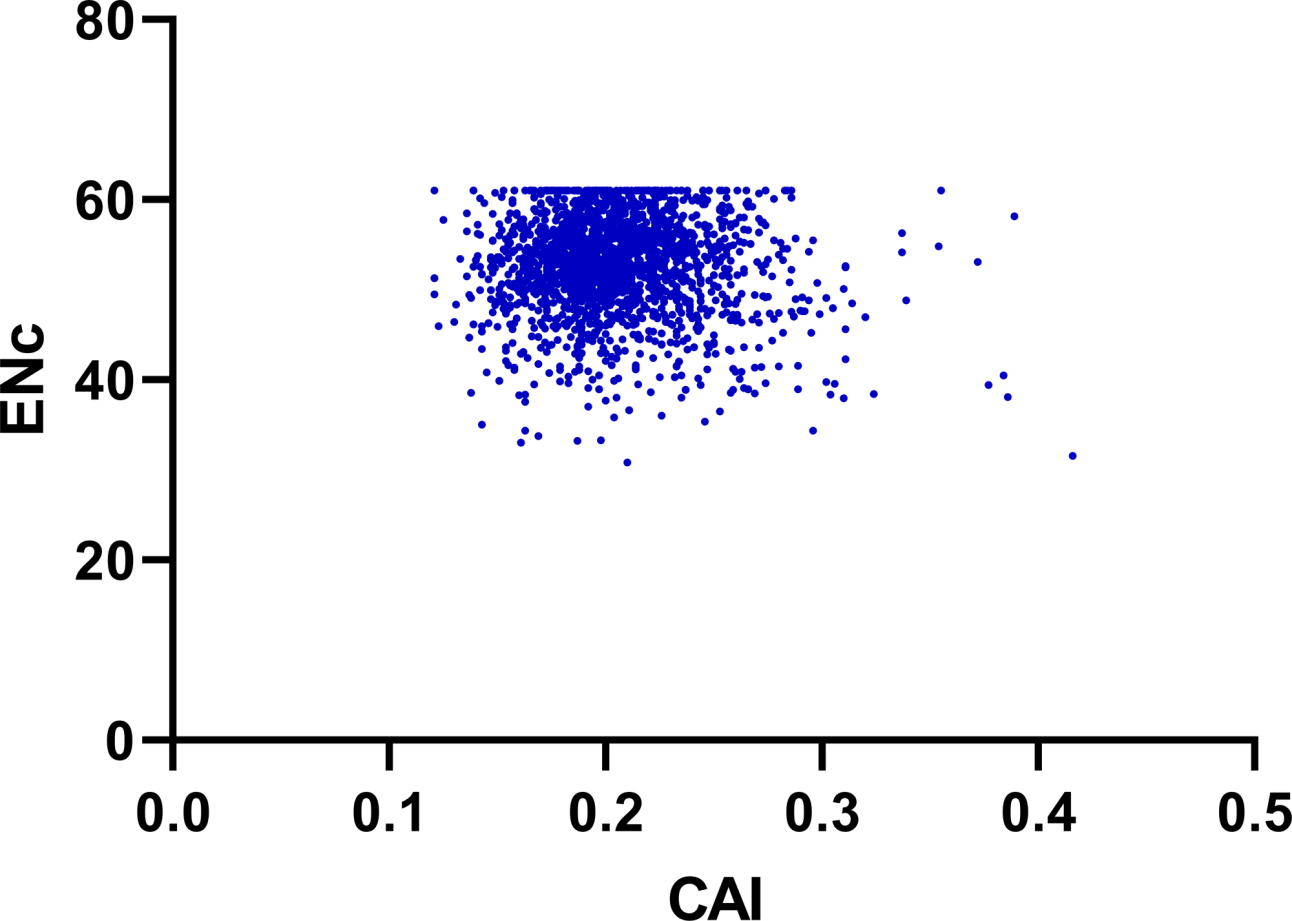
Neutrality plot (ENC vs. CAI).

To determine the effect of gene expression level in shaping codon bias, we performed correlation coefficients analysis between the codon adaptation index (CAI) values and index values of the genes including ENC, GC3, GC12 and GCall content (Guan et al., 2018). As shown in Fig. 7 and Table 2, the index CAI values indicated a significantly negative correlation (*r* = −0.054, *p* < 0.001) with the ENC values, and showed a remarkable positive correlation with GC3 (*r* = 0.232, *p* < 0.001), GC12 (*r* = 0.059, *p* < 0.001) and GCall (*r* = 0.176, *p* < 0.001) content.

### Determination of optimal codons for *R. palmatum*

We carried out a two-way Chi-squared contingency test to compare the codon usage among different genes. The highly- and lowly- expressed data of the genes based on average RSCU values were list in the Table 3. As shown in Table 3, 28 optimal codons were figured out and most of the optimal codons ended with A or U, except UUG for Leu. All the amino acids were coded by different codons such as Leu possess four codons and Ser possess three codons, Ile, Val, Arg, Ala, Thr and Pro all identified by two codons and others amino acids were all defined by only one codon. The results above indicated that the *R. palmatum* genes were preferred to A/U-ending synonymous codons which was inconsistent with *Triticum aestivum* (Zhang et al., 2007), *Oryza sativa* (Liu et al., 2004) and *Zea mays* (Liu et al., 2010) that preferred to G/C-ending synonymous. Our result was consistent with the study of codon usage patterns in *Lonicera macranthoides* which was also biased to A/U-ending synonymous codons (Liu et al., 2019).

**Table 3:** Optimal codons of *R. palmatum* genes based on the RSCU values.

Amino acid	Codon	High		Low		Amino acid	Codon	High		Low	
		RSCU	N	RSCU	N			RSCU	N	RSCU	N
Phe	UUU^*^	1.33	1118	0.42	265	Ser	UCU^*^	1.83	1103	0.77	338
	UUC	0.67	567	1.58	986		UCC	0.58	349	1.98	873
Lys	AAA^*^	0.98	1149	0.47	290		UCA^*^	1.62	976	0.51	225
	AAG	1.02	1193	1.53	956		UCG	0.24	146	1.13	500
Cys	UGU^*^	1.46	621	0.49	146		AGU^*^	1.11	669	0.38	166
	UGC	0.54	229	1.51	449		AGC	0.62	373	1.24	548
Leu	UUA^*^	1.14	631	0.25	110	Pro	CCU^*^	1.92	829	0.73	339
	UUG^*^	1.55	861	0.88	388		CCC	0.39	167	1.34	619
	CUU^*^	1.60	884	0.67	294		CCA^*^	1.55	666	0.49	228
	CUC	0.46	257	2.56	1132		CCG	0.14	62	1.43	660
	CUA^*^	0.59	325	0.37	162	Asn	AAU^*^	1.37	1231	0.50	260
	CUG	0.66	365	1.28	564		AAC	0.63	562	1.50	787
Asp	GAU^*^	1.52	1708	0.70	489	Trp	UGG	1.00	412	1.00	383
Ile	AUC	0.67	444	1.84	790	Thr	ACU^*^	1.74	846	0.62	247
	AUA^*^	0.81	542	0.46	199		ACC	0.52	252	1.82	731
	AUU^*^	1.52	1010	0.70	301		ACA^*^	1.55	752	0.49	195
Met	AUG	1.00	967	1.00	676		ACG	0.19	90	1.08	432
Tyr	UAU^*^	1.36	779	0.31	126	Ala	GCU^*^	1.73	1023	0.70	491
	UAC	0.64	365	1.69	689		GCC	0.42	250	1.80	1260
Gly	GGU^*^	1.30	724	0.53	338		GCA^*^	1.70	1005	0.45	318
Val	GUU^*^	1.75	1045	0.65	337		GCG	0.14	83	1.05	738
	GUC	0.51	304	1.42	735	Gln	CAA^*^	1.15	846	0.63	287
	GUA^*^	0.77	463	0.28	146		CAG	0.85	626	1.37	619
	GUG	0.97	579	1.65	858	Arg	CGU^*^	0.78	244	0.41	115
TER	UAA	1.11	49	1.09	48		CGC	0.23	71	1.45	407
	UAG	0.68	30	0.68	30		CGA	0.39	124	0.60	167
	UGA	1.20	53	1.23	54		CGG	0.21	67	1.24	347
His	CAU^*^	1.50	750	0.48	169		AGA^*^	2.71	852	0.68	191
	CAC	0.50	249	1.52	540		AGG	1.68	529	1.62	452

**Notes.**

RSCU Relative Synonymous Codon Usage High/Low highly- and lowly-expressed datasets N number of codons

Codon usage was compared using a chi-square test to identify optimal codons.

* Codons that occur significantly more often (*p* <0.01).

## Discussion

A lot of theories have supposed to clarify the origin of codon usage bias. The two main theories are neutral theory and the “selection–mutation–drift” model (Sharp & Li, 1986; Bulmer, 1988). Based on the neutral theory, the mutations occur in coding positions must be mutate neutral, thus lead to random selection of synonymous codons, while according to the “selection–mutation–drift” pattern, codon usage bias only informed the balance between selection favoring optimal codons and mutation–drift allowing persistence of non-optimal codons (Sharp & Li, 1986). In the genes with high expression levels, the selection makes a significant role in shaping the codon usage bias, while in the genes with low expression levels mutation–drift plays important role in determining codon usage (Kliman & Hey, 1994). But with the appearance of genome information of more species, it seems that these two theories are not enough to prove the characteristics of codon usage anymore. For example, in *Oryza sativa* codon usage is the result of nucleotide composition and expression level of each gene, as well as CDS length (Liu et al., 2004). In our study, the factors involved in shaping *R. palmatum* codon usage bias include the GC content, expression level of genes and natural selection as well as mutation pressure.

A previous study has found that there was no significant correlation between codon usage bias and gene expression level in mammals, which was inconsistent with our result that the codon bias in *R. palmatum* genes was also influenced by gene expression level (Karlin & Mrázek, 1996). But in rice, the genes with high expression levels always have strong variation in codon usage that is consistent with our results which indicates that the highly expressed genes are preferred to GC-rich in codon usage (Liu et al., 2004).

Previous studies have found that codon usage bias is not affected by nucleotide composition in *Chlamydomonas reinhardtii* (Naya et al., 2001) and *Echinococcus* spp. (Fernandez, Zavala & Musto, 2001) genomes that GC-rich in genomes. In *R. palmatum*, there is clear heterogeneity of codon usage among genes: *R. palmatum* favored the A/U-ending codons. Some previous studies have reported that the genes from dicot plant prefer the A/U-ending codons such as *Malus domestica* (Van Hemert & Berkhout, 2016), *Myrica rubra* (Li et al., 2016) and *Lonicera macranthoides* (Feng et al., 2013). The plant *R. palmatum* is a kind of dicot plant in general and our result was consistent with the data reported that shows an A/U-ending codons preference. Moreover, the ratio of A/U-ended codons and G/C-ended codons for the most frequently used codons is 22:8. The result was consistent with that of nucleotide composition above and the phenomenon was also similar to other species with richer AT contents, namely *Kluyveromyces lactis*, *Saccharomyces cerevisiae* and *Pichia pastoris* (Peixoto, Fernandez & Musto, 2004; Liu et al., 2019).

## Conclusions

Codon usage bias is a common and a complicated natural phenomenon for various kinds of living beings (Banerjee & Roy, 2009). We can illuminate the regular patterns of evolution, find out some new genes, optimize heterogeneous expression system through the overall codon usage bias analysis (Galtier et al., 2018). With the rapid development of high throughput sequencing, the analysis of codon usage bias pattern based on genome and transcriptome data is increasing rapidly (Angov, 2011; Goodman, Church & Kosuri, 2013). Such analysis based on big data is very useful to better understand evolutionary mechanisms of species and translation selection force in shaping codon usage bias.

In our project, we carried out the codon usage bias analysis of *R. palmatum* genes based on the transcriptome data. The GC content in *R. palmatum* CDS was 46.57%, which indicated that the CDSs of *R. palmatum* were slightly AT rich. Furthermore, the optimal codons analysis suggested that the *R. palmatum* CDS were preferred to A/U-ending synonymous codons. All these results clarified that the nucleotide composition of *R. palmatum* plays a significant role in shaping codon bias. Meanwhile, codon bias in *R. palmatum* genes was also influenced by gene expression level. In addition, 28 optimal codons were figured out and most of the optimal codons ended with A or U, except UUG for Leu. After a series of analyses, the codon usage bias in *R. palmatum* is influenced by nucleotide composition, natural selection, mutation pressure, and gene expression level.

In conclusion, our data offers new perspectives for the codon usage pattern in *R. palmatum* and has made a firm foundation for the gene engineering in *R. palmatum*.

## Additional Information and Declarations

### Data Availability

The following information was supplied regarding data availability: Data is available at NCBI SRA: SRX7526052.
